# Supplementary material for: Effect of p-TsOH pretreatment on separation of bagasse components and preparation of nanocellulose filaments
Source: R Soc Open Sci. 2020 Sep 9;7(9):200967. doi: 10.1098/rsos.200967 (PMC7540794; doi:10.1098/rsos.200967)
Supplement: Effect of p-TsOH dosage, temperature and time on the main component of bagasse [file rsos200967supp1.docx]

Effect of *p*-TsOH dosage, temperature and time on the main component of bagasse

ESM for Fig. 1

| *p*-TsOH dosage (%) | Glucose (%) | Xylan (%) | Lignin (%) |
| --- | --- | --- | --- |
| 40 | 46.69 | 19.81 | 21.33 |
| 50 | 45.32 | 16.84 | 15.43 |
| 60 | 44.27 | 14.11 | 10.06 |
| 70 | 43.37 | 10.89 | 6.33 |
| 80 | 42.86 | 6.85 | 3.17 |
| 90 | 42.66 | 4.08 | 2.42 |

ESM for Fig. 2

| Temperature (℃) | Glucose (%) | Xylan (%) | Lignin (%) |
| --- | --- | --- | --- |
| 50 | 46.39 | 19.04 | 21.17 |
| 60 | 44.78 | 15.47 | 15.37 |
| 70 | 43.84 | 10.76 | 9.87 |
| 80 | 43.06 | 6.49 | 2.45 |
| 90 | 42.81 | 4.34 | 2.21 |
| 100 | 42.70 | 3.55 | 1.93 |

ESM for Fig. 3

| Time (min) | Glucose (%) | Xylan (%) | Lignin (%) |
| --- | --- | --- | --- |
| 10 | 45.19 | 15.99 | 14.6 |
| 20 | 42.96 | 6.49 | 2.53 |
| 30 | 42.22 | 4.23 | 2.33 |
| 40 | 41.86 | 3.03 | 2.17 |
| 50 | 41.47 | 2.77 | 1.97 |
| 60 | 41.19 | 2.36 | 1.89 |
